# Chairmen's Reports of Provincial Hospitals

**Published:** 1919-12-20

**Authors:** 


### CHAIRMEN'S REPORTS OF PROVINCIAL HOSPITALS.

### BEDFORDSHIRE.

### BEDFORD COUNTY HOSPITAL.

(*a*) Immediate Needs.

*What we asked for this year:*
£8,000 for hospital maintenance (before the war £5,000 annually sufficed). £8,000 for a convalescent home of twenty beds.

*The Answer to December 11th:*
£8,000 for hospital maintenance. £7,200 for a convalescent home.

(*b*) Needs for 1920.

The rebuilding of Nurses' Home (£3,300 already in hand). The rebuilding of mortuary. The opening of convalescent home.

(*Signed*) J. Arnold Whitchurch, *Chairman.*

### BERKSHIRE.

### ROYAL BERKSHIRE HOSPITAL, Reading.

(*a*) Immediate Needs.

£6,000 to meet the deficit on general account, 1919.

(*b*) Needs for 1920.

Increased support to the amount of about £6,000 a year.

(*Signed*) H. Thornton, *Chairman.*

### KING EDWARD VII. HOSPITAL, Windsor.

(*a*) Immediate Needs.

£3,000 to clear debt on year's working, 1919.

(*b*) Needs for 1920.

Nurses' Home for fifty beds. New ward for sixty beds.

(*Signed*) William Shipley, *Vice-Chairman.*

### CORNWALL.

### ROYAL CORNWALL INFIRMARY.

(*a*) Immediate Needs.

Increased income to meet annual expenditure. Deficit, May 1918-19, £374; estimated, May 1919-20, £400.

(*b*) Needs for 1920.

A fund is also being raised for extensions and improvements, towards which £4,172 has been received. A further £328 is required to obtain £4,500 grant from the British Red Cross Society. Owing to increase in prices £11,000 will be required for scheme.

(*Signed*) W. E. Laon, *Chairman.*

### CHESHIRE.

### CHESTER ROYAL INFIRMARY.

(*a*) Immediate Needs.

Gift to liquidate the debt—new buildings and alterations, £10,000; general account, £9,000; total £19,000; *less* special thanksgiving appeal, £5,400; total, £13,600.

(*b*) Needs for 1920.

(1) Gifts of £500 for endowment of cots in the new children's ward, which is being completely furnished and decorated by two generous friends of the Charity. (2) New annual subscriptions. (3) Additional contributions to the Hospital Saturday Fund.

(*Signed*) E. M. Sneyd Kynnersley, *Chairman.*

### GENERAL INFIRMARY, Macclesfield.

(*a*) Immediate Needs.

Increased annual subscriptions.

(*b*) Needs for 1920.

A Nurses' Home.

(*Signed*) A. E. Harnahan (for Chairman).

### DEVONSHIRE.

### ROYAL DEVON AND EXETER HOSPITAL, Exeter.

(*a*) Immediate Needs.

Additional special contributions to meet adverse balance of £3,000 at the end of 1919.

(*b*) Needs for 1920.

Extra contributions towards the contemplated extension of a hundred beds, fifty to be set aside entirely for in-patient treatment for discharged sailors and soldiers, and the remaining fifty for treatment of child-welfare cases. The British Red Cross Society has promised £10,000 towards these improvements, and the Treasury, H.M. Pensions, £2,000.

(*Signed*) E. Chaning Wills, Bt., *Chairman.*

### SOUTH DEVON AND EAST CORNWALL HOSPITAL, Plymouth.

(*a*) Immediate Needs.

£7,000 to debt, end of 1919.

(*b*) Needs for 1920.

£4,000 to maintenance. £2,350 for new clinic, massage, electro-therapeutic, bio-chemical, and investigation and *x*-ray departments; laundry and site, £5,000.

### DURHAM.

### THE ROYAL INFIRMARY, Sunderland.

(*a*) Immediate Needs.

£3,000 to defray debt.

(*b*) Needs for 1920.

1. Additional income of £7,000 to meet annual expenditure. 2. Reduction of hours and increased rates of pay for nursing staff. Cost: Capital, £1,000; annual expenditure, £1,000. 3. New administrative block. Present one built for 96 beds, now quite inadequate for 236 beds; estimated cost, about £120,000. 4. Special departments needed: (*a*) Ear, nose, and throat; (*b*) pathological; (*c*) dental; (*d*) additional operation theatre; (*e*) additional appliances for electrical department.

(*Signed*) S. C. Fryers, *Secretary.*

### ESSEX.

### ESSEX COUNTY HOSPITAL, Colchester.

(*a*) Immediate Needs.

£5,000 per annum to meet increased prices, salaries and wages. A special appeal has so far realised increased annual subscriptions £706, donations £3,209, whilst Hospital Saturday and workers' collection have substantially increased.

(*b*) Needs for 1920.

Increased income exceeding 1919, and additional accommodation. The latter is under consideration in connection with the centenary celebration in 1920. Plans have been prepared, and the present estimated cost will be about £150,000. Reconstruction to extend over some years.

(*Signed*) Reginald D. Hill, *Chairman.*

### GLOUCESTERSHIRE.

### CHELTENHAM GENERAL HOSPITAL

(*a*) Immediate Needs.

£5,000 to £6,000 to clear off deficiency.

(*b*) Needs for 1920.

New out-patient department; £6,000 promised on certain conditions by British Red Cross Society.

(*Signed*) R. H. M. Bouth, *Chairman.*

### HAMPSHIRE.

### ROYAL VICTORIA AND WEST HANTS HOSPITAL, Bournemouth.

(*a*) Immediate Needs.

£10,000 to extinguish floating debt.

(*b*) Needs for 1920.

Sound basis for annual income; nurses' home wanted very badly (£10,000 in hand towards this); central heating and power station; addition to laundry.

(*Signed*) E. H. Bellairs, *Chairman.*

### ROYAL PORTSMOUTH HOSPITAL.

(*a*) Immediate Needs.

£5,000 to pay off debt to bankers; £5,000 additional income, annual subscriptions, and workpeople's weekly contributions.

### CHAIRMEN'S REPORTS OF PROVINCIAL HOSPITALS—(continued.)

(a) Immediate Needs.

New buildings, including an additional ward block, pathological department, and laundry.

(Signed) Harold Pink [Knight], Chairman.

### ROYAL HANTS COUNTY HOSPITAL, Winchester.

(a) Immediate Needs.

Sanitary accommodation for nursing staff, £1,000; regravelling front drive, £250.

(b) Needs for 1920.

To collect a large sum to carry out building scheme to modernise the hospital in every department, especially the better housing of resident medical officers and the domestic staff.

(Signed) Charles W. Oddie, Chairman.

### KENT.

### GRAVESEND HOSPITAL, Kent.

(a) Immediate Needs.

To pay off debt of £1,500.

(b) Needs for 1920.

Extension to double accommodation owing to increasing work.

(Signed) H. D. Stephenson, Chairman.

### SAINT BARTHOLOMEW'S HOSPITAL, Rochester.

(a) Immediate Needs.

Central heating installation; modernised kitchen department; enlarged casualty department.

(b) Needs for 1920.

£2,000 new income (£1,000 to meet increased expenditure, and £1,000 to make good lost income).

(Signed) H. d'Arch Breton [Colonel], Chairman.

### GENERAL HOSPITAL, Tunbridge Wells.

(a) Immediate Needs.

A larger annual income.

(b) Needs for 1920.

An extension of in-patient accommodation; a further 50 beds are urgently needed. £25,000 is being asked for to commence the building

(Signed) J. S. Snelgrove, Chairman.

### LANCASHIRE.

### DISTRICT INFIRMARY, Ashton-under-Lyne.

(a) Immediate Needs.

£14,000 to extinguish the debt on maintenance and extension funds.

(b) Needs for 1920.

At least £50,000 to carry out extensions and improvements which had to be postponed when war commenced. (Signed) Albert Shaw, Chairman.

### BURY INFIRMARY, Bury, Lancashire.

(a) Immediate Needs.

Greatly increased subscriptions and donations to meet expenses. The provision of a children's ward and twelve additional bedrooms to meet the requirements of extra staff, which will be necessary in adopting shorter hours, as recommended by the College of Nursing.

(b) Needs for 1920.

A new out-patients' department and receiving-rooms; estimated cost, £15,000 to £20,000.

(Signed) S. H. Renshaw, Chairman.

### LIVERPOOL ROYAL INFIRMARY.

(a) Immediate Needs.

New Nurses' Home of 150 bedrooms; present Home inadequate and out of date.

(b) Needs for 1920.

Also increased income to meet extra cost of commodities and upkeep generally.

(Signed) H. Wade Deacon, Chairman.

### DAVID LEWIS NORTHERN HOSPITAL, Liverpool.

(a) Immediate Needs.

£11,000 to pay off debt.

(b) Needs for 1920.

£4,000 increased income; extension of Nurses' Home at a cost of about £6,500.

(Signed) M. Scott Barrett, Chairman.

### ROYAL LIVERPOOL CHILDREN'S HOSPITAL.

(a) Immediate Needs.

To clear off debt; increased subscriptions and donations.

(b) Needs for 1920.

£70,000 to complete the hospital by adding two wings to accommodate 140 more children, and to enlarge the out-patient department.

(Signed) A. Hyslop Mansell, Chairman.

### MANCHESTER ROYAL EYE HOSPITAL.

(a) Immediate Needs.

To pay off bank overdraft.

(b) Needs for 1920.

New out-patient department; improved pathological arrangements.

(Signed) T. W. Kessler, Chairman.

### OLDHAM ROYAL INFIRMARY.

(a) Immediate Needs.

Increased accommodation for nurses; new out-patients' department, including medical, surgical, ophthalmic, ear, nose and throat, dental, massage, x-ray and electro-therapeutic, and venereal departments; pathological and bacteriological laboratory; additional beds for men and women; new arrangement for heating the whole infirmary by steam; a destructor.

(Signed) Thomas Fawsitt, Chairman.

### PRESTON AND COUNTY OF LANCASTER ROYAL INFIRMARY.

(a) Immediate Needs.

No serious deficit on maintenance account for 1919; expenditure £3,000 higher than 1918, but expected to be met by increased subscriptions, donations, collections, payments for pensioners, patients' payments, and first Alexandra Day (£603).

(b) Needs for 1920.

£1,000 additional income needed for extra nurses in connection with recently started shorter hours (one day off in seven) and for increased salaries. Capital: £1,500 for new ward to hold 20 beds decided to be erected to meet demand for beds; £1,200 for additions to Nurses' Home in course of erection; £2,000 for alterations and furnishing of "Lostock Hall," recently presented for use as a convalescent home; £2,000 for orthopædic department; £400 for improvements to laundry. (Signed) J. C. Hamilton, Chairman.

### ROCHDALE INFIRMARY AND DISPENSARY.

(a) Immediate Needs.

Increased income to clear off the heavy debit balance, mainly due to the ever-increasing cost of upkeep and maintenance.

(b) Needs for 1920.

The adoption of other systematic methods (apart from those already in use) for increasing the annual income of the institution, if it is to retain its efficiency. New schemes are contemplated.

### SOUTHPORT GENERAL INFIRMARY, Scarisbrick New Road, Southport.

(a) Immediate Needs.

1. Increased income to meet the growing expenditure. 2. Deficit on general account of £7,000 should be liquidated. 3. New out-patients' department, as present one totally inadequate to cope with heavy demands mainly by discharged and disabled soldiers and sailors.

(b) Needs for 1920.

1. Increased income from all sources. 2. New out-patients' department.

(Signed) for Sir J. W. Paton, J.P., Chairman.

### CHAIRMEN'S REPORTS OF PROVINCIAL HOSPITALS—(*continued.*)

### WARRINGTON INFIRMARY.

(*a*) Immediate Needs.

£4,000 for extensions to Nurses' Home, owing to increased staff to give shorter hours for staff.

(*b*) Needs for 1920.

General increased subscriptions. Just had a special appeal and raised £10,000 for past deficiencies and to provide a little surplus.

(*Signed*) Jas. Hy. Smethurst, *Chairman.*

### LEICESTERSHIRE.

### LEICESTER ROYAL INFIRMARY.

(*a*) Immediate Needs.

(1) £45,000 for a new wing (ninety-six beds). (2) £36,000 for additions to Nurses' Home. (3) £1,200 for alterations to laundry.

(*b*) Needs for 1920.

Additions to site area. Buildings in course of construction: Orthopædic O.P. Department; Pathological Department, £10,000. New Mortuary; Viewing Room Chapel. Total cost £12,000.

### MONMOUTHSHIRE.

### ROYAL GWENT HOSPITAL, Newport, Mon.

(*a*) Immediate Needs.

New O.P Department. British Red Cross Gift £10,000.

(*b*) Needs for 1920.

Increased accommodation (1) for in-patients, (2) for nursing staff, costing £15,000.

### NORFOLK.

### NORFOLK AND NORWICH HOSPITAL.

(*a*) Immediate Needs.

Central heating, £15,000. An overdraft for the year of about £8,000.

(*Signed*) James Barton, *Chairman.*

### NORTHAMPTONSHIRE.

### NORTHAMPTON GENERAL HOSPITAL.

(*a*) Immediate Needs.

Central heating scheme; new kitchens; increased accommodation for nurses.

(*b*) Needs for 1920.

New isolation block; hospital chapel.

(*Signed*) H. Manfield, *Chairman.*

### NORTHUMBERLAND.

### ROYAL VICTORIA INFIRMARY, Newcastle-on-Tyne.

(*a*) Immediate Needs.

Deficit on income for 1919, £30,000 (approximate); necessary capital expenditure, either incurred or immediately necessary, £15,000.

(*b*) Needs for 1920.

Extension to Nurses' Home, £110,000; wards, £50,000; pathological block, £50,000; the maintenance of 400 additional beds (at present used for soldiers or pensioners), which will involve an additional annual expenditure of £52,000.

(*Signed*) R. Orde (for Chairman).

### NOTTINGHAMSHIRE.

### MANSFIELD AND DISTRICT HOSPITAL.

(*a*) Immediate Needs.

£5,000 to pay off overdraft for that amount. It is hoped to raise this sum by a Shilling Fund and House-to-House Collection on Christmas Day.

(*b*) Needs for 1920.

Increased income to meet increased expenditure. It is proposed to obtain the necessary money by increased contributions from the workpeople in the district covered by the hospital.

(*Signed*) W. F. Jolly, *Chairman.*

### NOTTINGHAM GENERAL HOSPITAL.

A Splendid Effort.

(*a*) Immediate Needs.

Double present income to defray increased expenditure. Required £40,000 per year instead of £20,000. War Memorial £92,000, raised £8,000. More required to pay for New Nurses' Home.

(*b*) Needs for 1920.

As stated in (*a*) already workpeople (led by the miners) are doubling their weekly contribution. That class will find their extra £10,000 yearly, and it is hoped that middle and upper classes, "helping those who are thus helping themselves," will find the other half needed.

(*Signed*) Frederick Acton, *Chairman.*

### SHROPSHIRE.

### ROYAL SALOP INFIRMARY, Shrewsbury.

(*a*) Immediate Needs.

£5,000 to liquidate our debt to the bank and to maintain the hospital in full efficiency.

(*b*) Needs for 1920.

Provision of accommodation for from thirty to forty children and maternity cases needing special attention.

(*Signed*) Alfred Mansell, *Chairman.*

### SOMERSET.

### ROYAL UNITED HOSPITAL, Bath.

(*a*) Immediate Needs.

£4,000 to meet expenditure for 1919. If the ordinary income is not increased in 1920 a similar amount will be required next year to balance the annual account.

(*b*) Needs for 1920.

A hospital for paying patients. A scheme is under consideration for considerable extension to meet the growing demands for improvement in the investigation and treatment of disease.

(*Signed*) Egbert Lewis [Lt.-Col.], *Chairman.*

### STAFFORDSHIRE.

### WALSALL GENERAL HOSPITAL.

(*a*) Immediate Needs.

Largely increased income: £3,000 from workpeople instead of £2,000; £2,000 from subscribers instead of £700.

(*b*) Needs for 1920.

A large sum to enable the committee to enlarge hospital to 300 beds—*i.e.*, two per 1,000 of population served. Present beds only 90.

(*Signed*) W. G. Francombe, *Secretary* (for Chairman).

### WEST BROMWICH AND DISTRICT HOSPITAL.

(*a*) Immediate Needs.

£4,000 to wipe off debt. £5,000 for enlargement of Nurses' Home and Out-patient Department.

(*b*) Needs for 1920.

£1,500 increase annual income.

(*Signed*) Wm. Thos. Davies, *Chairman.*

### WOLVERHAMPTON AND STAFFS GENERAL HOSPITAL.

(*a*) Immediate Needs.

A boiler-house and laundry.

(*b*) Needs for 1920.

£150,000 for the reconstruction scheme.

(*Signed*) Tom B. Adams, *Chairman.*

### SUFFOLK.

### WEST SUFFOLK HOSPITAL, Sudbury.

(*a*) Immediate Needs.

£6,000 to meet similar sum given by Red Cross Society to build Nurses' Home, separate laundry, *x*-ray department, kitchen, and instal central heating.

(*b*) Needs for 1920.

An immediate increase in revenue of at least £2,000 to meet expenditure which has doubled itself since 1914.

(*Signed*) Edward W. Lake, *Chairman.*

### CHAIRMEN'S REPORTS OF PROVINCIAL HOSPITALS—(*continued.*)

### SURREY.

**ROYAL SURREY COUNTY HOSPITAL, Guildford.**

(*a*) Immediate Needs.

Sums of £1,500 to meet cost of re-roofing hospital and £1,500 to cover overdraft at bankers.

(*b*) Needs for 1920.

Large capital sum for new buildings in order to carry out scheme of special departments recommended by hon. medical staff.

(*Signed*) FERDINAND SMALLPEICE, *Chairman.*

### WARWICKSHIRE.

**BIRMINGHAM AND MIDLAND HOSPITAL FOR WOMEN (Incorporated).**

(*a*) Immediate Needs.

£4,000 to wipe out accrued deficit during the year.

(*b*) Needs for 1920.

£5,000 increased income to enable the hospital to keep free from debt.

(*Signed*) HUGH C. ALLEN, *Hon. Sec*

**COVENTRY AND WARWICKSHIRE HOSPITAL, Coventry.**

(*a*) Immediate Needs.

To clear off debt on Extension Fund, £14,000. Deficit on working expenses 1919, £7,000. Total, £21,000.

(*b*) Needs for 1920.

Increased income of £14,000. £12,000 to carry out immediate extensions.

(*Signed*) W. J. WORSNELL, *Chairman.*

**WARNEFORD GENERAL HOSPITAL, Leamington Spa.**

(*a*) Immediate Needs.

Enlargement of Nurses' Home, owing to increase of nursing staff due to decreased hours and new departments.

(*b*) Needs for 1920.

Rebuilding and reconstruction of out-patients' department. Build maternity wards and special wards for ophthalmic, ear, nose, and throat departments.

(*Signed*) FREDERICK SHAW, *Chairman.*

### WILTSHIRE.

**SALISBURY INFIRMARY.**

(*a*) Immediate Needs.

£5,000 to pay off some of our debt.

(*b*) Needs for 1920.

Another £5,000 to put us in credit.

(*Signed*) E. D. H. BUCKLEY [Major] *Chairman.*

### WORCESTERSHIRE.

**THE GUEST HOSPITAL AND EYE INFIRMARY, Dudley, Worc.**

(*a*) Immediate Needs.

Clearance of an accumulated debit balance of £6,500. £2,000 additional annual income.

(*b*) Needs for 1920.

A new Nurses' Home. Our nursing staff has increased and the sleeping accommodation is very inadequate. £2,000 additional income.

(*Signed*) ARTHUR BIRD, *for Chairman.*

### YORKSHIRE.

**BECKETT HOSPITAL, Barnsley.**

(*a*) Immediate Needs.

£15,000 amount of deficit accumulated by war-time increased cost. Also additional accommodation for medical cases. (*b*) Needs for 1920.

Increased subscriptions to meet increased cost. At present income is about £4,000 to £5,000 short of what is required annually.

(*Signed*) ALFRED WHITHAM, *Chairman.*

**ROYAL INFIRMARY, Bradford.**

(*a*) Immediate Needs.

£15,000 to clear off sum owing to bankers.

(*b*) Needs for 1920.

£10,000 additional annual income. £500,000 to erect new general hospital.

(*Signed*) WM. ERNEST TETLEY, *Chairman.*

**GENERAL INFIRMARY AT LEEDS.**

(*a*) Immediate Needs.

Overdraft at bank: On General Fund, £32,000; on King Edward Fund, £9,000; total, £41,000.

(*b*) Needs for 1920.

Permanent increase of income, £30,000; to complete King Edward Memorial Scheme, £20,000.

**NORTH ORMESBY HOSPITAL, Middlesbrough.**

(*a*) Immediate Needs.

£10,000 for extension of buildings for nurses, shorter hours, and higher pay.

(*b*) Needs for 1920.

Enlargement of hospital to meet growing needs of district. Nurses' Home to accommodate more nurses. Present staff are overworked.

(*Signed*) J. W. PENNYMAN, *Chairman.*

**THE ROYAL INFIRMARY, SHEFFIELD.**

(*a*) Immediate Needs.

An increased annual income. Another £5,000 in annual subscriptions, and £5,000 more from the workpeople of the city. The expenditure this year will be £10,000 in excess of income.

(*b*) Needs for 1920.

£12,000 to complete the £100,000 appeal. Increased subscriptions and workmen's contributions. Extensions in the out-patient department. A joint Hospitals Advisory Committee, comprising representatives of all the hospitals, is considering the position of the Sheffield voluntary hospitals, and it is sincerely hoped that the voluntary principle will long continue. (*Signed*) H. H. BEDFORD, *Chairman.*

**THE SHEFFIELD ROYAL HOSPITAL.**

(*a*) Immediate Needs.

£12,000, being the balance of a joint appeal by this hospital and the Royal Infirmary, Sheffield, for £100,000.

(*b*) Needs for 1920.

An increased annual income. £6,000 to meet deficit.

(*Signed*) PHILIP R. WAKE, *Chairman.*

### Scotland.

**THE ROYAL INFIRMARY OF EDINBURGH.**

(*a*) Immediate Needs.

£20,000 to wipe out the deficit on the past year's accounts.

(*b*) Needs for 1920.

An increase of £40,000 on the ordinary income. A renewal and reconstruction fund of £100,000 to meet the cost of repainting, machinery renewals, kitchen extension, etc.

(*Signed*) G. L. CROLE, LL.D., K.C. [Sheriff] *Chairman.*

**WESTERN INFIRMARY OF GLASGOW (Incorporated).**

(*a*) Immediate Needs.

Increased and additional annual subscriptions for maintenance. Deficit for year ended October 31 last, £27,719.

(*b*) Needs for 1920.

Ordinary expenditure estimated at, say, £86,000; ordinary income (say), £53,000. Deficit, £33,000. To meet this deficit the income from subscriptions will require to be doubled.

(*Signed*) J. MATHIESON JOHNSTON, *Secretary & Treasurer.*

### PARTICULARS OF SCOTTISH HOSPITALS—(*continued.*)

### GLASGOW ROYAL INFIRMARY.

(*a*) Immediate Needs.

Estimated deficiency for 1919, £47,160.

(*b*) Needs for 1920.

£35,000 additional income.

### STIRLING ROYAL INFIRMARY.

(*a*) Immediate Needs.

£1,000 more income on maintenance account.

(*b*) Needs for 1920.

£2,000 maintenance account. Expenditure for 1920 is likely to be even larger than for the year ending October 31, 1919, whereas our income will be less, as we will miss the grant from the military authorities for the treatment of military patients.

### Ireland.

### MEATH HOSPITAL AND CO. DUBLIN INFIRMARY, Dublin.

(*a*) Immediate Needs.

Debit bank balances to-day £17,377 15s. 10d. Deficiency on account for year March 31, 1919, £2,324 18s. 2d.

(*b*) Needs for 1920.

Urgent need of increased support.

(*Signed*) L. H. ORMSBY [Sir], M.D., J.P., D.L., *Chairman.*

