# Erratum

**Published:** 1919-12-27

**Authors:** 


### Erratum.

West Suffolk Hospital, Bury St. Edmunds.—We regret that in our issue of last week, page 273, the address of the above hospital was wrongly given as Sudbury.

